# The validation of Fibit Zip™ physical activity monitor as a measure of free-living physical activity

**DOI:** 10.1186/1756-0500-7-952

**Published:** 2014-12-23

**Authors:** Mark A Tully, Cairmeal McBride, Leonnie Heron, Ruth F Hunter

**Affiliations:** Centre for Public Health, School of Medicine, Dentistry and Biomedical Sciences, Queens’ University Belfast, Belfast, UK; UKCRC Centre of Excellence for Public Health (Northern Ireland), Belfast, UK

**Keywords:** Physical activity, Pedometer, Accelerometer, Validation

## Abstract

**Background:**

The new generation of activity monitors allow users to upload their data to the internet and review progress. The aim of this study is to validate the Fitbit Zip as a measure of free-living physical activity.

**Findings:**

Participants wore a Fitbit Zip, ActiGraph GT3X accelerometer and a Yamax CW700 pedometer for seven days. Participants were asked their opinion on the utility of the Fitbit Zip. Validity was assessed by comparing the output using Spearman’s rank correlation coefficients, Wilcoxon signed rank tests and Bland-Altman plots. 59.5% (25/47) of the cohort were female. There was a high correlation in steps/day between the Fitbit Zip and the two reference devices (r = 0.91, p < 0.001). No statistically significant difference between the Fitbit and Yamax steps/day was observed (Median (IQR) 7477 (3597) vs 6774 (3851); p = 0.11). The Fitbit measured significantly more steps/day than the Actigraph (7477 (3597) vs 6774 (3851); p < 0.001). Bland-Altman plots revealed no systematic differences between the devices.

**Conclusions:**

Given the high level of correlation and no apparent systematic biases in the Bland Altman plots, the use of Fitbit Zip as a measure of physical activity. However the Fitbit Zip recorded a significantly higher number of steps per day than the Actigraph.

## Findings

### Introduction

The use of physical activity monitors have been shown to be an effective means to promote changes in physical activity [1]. These have evolved over time from relatively simple mechanical pedometers (that display but not record physical activity in steps), to more complex accelerometers (that record intensity and duration of movement). There has been a recent proliferation of a new generation of physical activity monitors that use accelerometer type mechanisms, but with a much simpler, user friendly interface, thus overcoming the need for technical expertise in analysing and reporting outcomes. These devices are produced by mainstream commercial companies and are designed to allow the user to upload their physical activity record to a user’s account via the internet or mobile phone application, allowing the user to review progress and share via social media.

Internet delivered physical activity interventions have emerged as an attractive option for health promotion, due to its potentially wide population reach [2]. In a systematic review, Davies et al. [3] demonstrated that internet delivered physical activity interventions are effective at producing small changes in physical activity. They recommended that future research should use validated instruments to measure study outcomes. Much of the previous research has involved the validation of energy expenditure in controlled environments [4–7]. There is therefore a need to assess the validity of this new generation of physical activity monitors as measures of free-living physical activity. One such device is the Fitbit Zip™ physical activity monitor which is a relatively cheap ($60US) step counter that can wirelessly upload data to the users account via a USB dongle connected to their PC or a range of mobile applications. The aim of this study is to test the validity of the Fitbit Zip™ as a measure of free-living physical activity.

### Methods

#### Participants

Staff employed in the School of Medicine, Dentistry and Biomedical Science, Queen’s University Belfast were invited to participate via email. Individuals were encouraged to respond via email to indicate their willingness to participate. Participants were eligible to take part if they reported no known disease or injury that would prevent them taking regular physical activity and were willing to monitor their activity for a seven day period.

As previous data does not exist to inform a sample size calculation, it was planned to recruit a convenience sample of 50 participants. This is comparable to the sample size used in previous validation studies of physical activity monitors [8, 9].

#### Data collection

All individuals who gave fully informed consent to participate were asked to wear a Fitbit Zip™ (Fitbit Inc, USA), with an ActiGraph GT3X accelerometer (Actigraph Inc, USA) and a Yamax CW700 pedometer (Yamax Inc, Japan). The Fitbit Zip™ devices were bought from an from an online retailer and not through the manufacturer. The manufacturers of the Fitbit Zip™ device had no role in the funding, design or conduct of the study, or analysis of the results.

Participants were asked to wear the device on their waist, at the right hand side. Participants were advised to remove the devices during water activities and sleeping. They completed a daily diary to record periods when the devices were removed.

After seven days, individuals were invited to return the devices and diary. Demographic information such as age and gender were recorded, and participants were asked for their written feedback on Fitbit Zip™, using a utility questionnaire adapted from previous research [10].

#### Data handling

Data was cleaned by removing non-wear time for the pedometers and Actigraph accelerometer by referring to the wear time diary. At least five days of valid data were required for the Actigraph data to be included in the analysis. A valid day was defined as a 24 hour period in which at least 10 hours of data wear time was recorded. Non-wear time was analysed as a run of zero counts lasting more than 60 minutes [11].

Data from the Fitbit Zip was recorded from the internet log of steps per day. The Yamax pedometer has a 7-day memory and this was accessed and the steps/day recorded in an electronic spreadsheet. These were both conducted by the researcher at the end of the 7-day wear period and average steps/day was calculated. At the end of the study, the Actigraph data was analysed using Actilife 6.0 (Actigraph Inc, USA) to calculate average steps/day and minutes of moderate or vigorous physical activity (MVPA) per day using Freedson cutpoints (>1952 counts/min) [12].

#### Data analysis

Statistical analysis was performed using SPSS (Version 21). Descriptive statistics were calculated for each variable. As the Actigraph data was non-normally distributed, appropriate non-parametric statistical tests were used.

The validity of Fitbit Zip as a measure of free-living physical activity was assessed by comparing it’s output (steps/day) with that of the Actigraph accelerometer (steps/day and mins of MVPA) and Yamax pedometer (steps/day), according to the recommendations to Welk et al. [13] Firstly, to ascertain if the output from the Fitbit Zip™ was associated with that of the two reference devices, Spearman’s rank correlation coefficients were calculated. To assess if the output from the devices yielded similar group estimates, the differences between the Fitbit Zip™ and the reference devices was assessed using Wilcoxon signed rank tests. Finally, Bland-Altman plots [14] were created to assess the level of agreement between the devices. The School of Medicine, Dentistry and Biomedical Sciences Research Ethics Committee, Queen’s University, Belfast approved the study (October 2013; Ref: 13.32v2).

### Results

Of the 582 people invited to participate by email, 48 (8%) agreed to take part. One of these individuals did not start the programme. At the end of the one week recording period, valid data was available for 89% (n = 42/47) of those who participated. The characteristics of the cohort are provided in Table 1. 59.5% (n = 25) of the cohort were female.

**Table 1:** **Descriptive characteristics of the cohort (n = 42)**

Measure	Median (IQR)
Age	43 (24)
Fitbit measured steps per day	7477 (3597)
Actigraph measured steps per day	6774 (3851)
Yamax measured steps per day	7532 (4105)
Actigraph measured MVPA (mins/day)	42.23 (27.19)

Comparing the Fitbit Zip™ with the two reference devices demonstrated high correlation with steps/day measured with both the Actigraph accelerometer and Yamax pedometer (both r = 0.91) and MVPA measured with the Actigraph device (r = 0.86) (Table 2). No statistically significant difference between the Fitbit and Yamax was observed, however the Fitbit measured significantly more steps/day than the Actigraph (7477 vs 6774; p < 0.001) (Table 2).

**Table 2:** **Comparison of the Fitbit Zip™ with the Actigraph and Yamax devices (n = 42)**

	Spearman correlation		Wilcoxon signed rank test
	***r***	***p-value***	***p-value***
Fitbit vs Actigraph (steps/day)	0.91	<0.001	<0.001
Fitbit vs Yamax (steps/day)	0.91	<0.001	0.11
Fitbit (steps/day) vs Actigraph (MVPA mins/day)	0.86	<0.001	-*

*It was not possible to compare differences between Actigraph measured MVPA with Fitbit steps/day as they are in different units.

**Figure 1:**
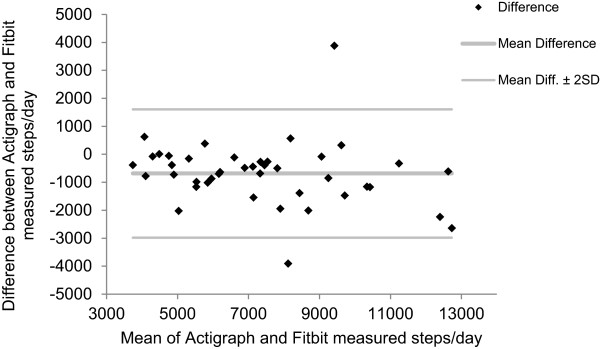
**Bland-Altman plot for Fitbit vs Actigraph steps/day (n=42).**

**Figure 2:**
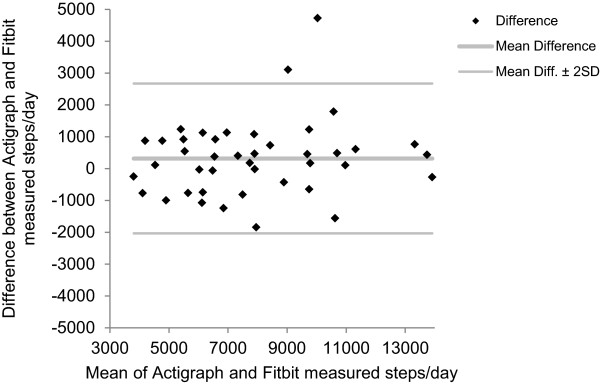
**Bland-Altman plot for Fitbit vs Yamax steps/day (n=42).**

Bland-Altman plots revealed no systematic differences between the Fitbit Zip™ and Actigraph (Figure 1) or Yamax devices (Figure 2) measured steps/day. Overall there was a high acceptability of the Fitbit Zip™. Although only one participant had used a Fitbit Zip™ previously, the majority of respondents rated the Fitbit Zip™ as acceptable to use and easy to integrate into their daily routine (Table 3).

**Table 3:** **Utility of the Fitbit Zip**™ **physical activity monitor**

Question	Response
Have you ever used a Fitbit to measure physical activity in the past?	Yes 2.4% (n = 1)
	No 97.6% (n = 41)
Was using the Fitbit every day for 7 days an acceptable method to measure your daily activity?	No acceptable (n = 0)
	Neither 21.4% (n = 9)
	Very acceptable 78.6% (n = 33)
How easy was it to remember to use the Fitbit Zip every day?	Difficult to remember 2.4% (n = 1)
	Neither 9.5% (n = 4)
	No problem 88.1% (n = 37)
Did using the Fitbit interfere with your daily routine?	Interfered greatly (n = 0)
	Neither 11.9% (n = 5)
	Did not interfere at all 88.1% (n = 37)
Was the Fitbit annoying to use?	Extremely annoying (n = 0)
	Neither 16.7% (n = 7)
	Not annoying at all 83.3% (n = 35)
Would you wear the Fitbit again as part of a research study?	No 0% (n = 0)
	Maybe 9.5% (n = 4)
	Yes 90.5% (n = 38)

### Discussion

The results indicate that the Fitbit Zip™ is a valid measure of physical activity. There is a significant correlation between the Fitbit Zip™ measured steps/day with that of both the mechanical pedometer (Yamax) and the Actigraph accelerometer. Given the high level of correlation and no apparent systematic biases in the Bland Altman plots, the use of Fitbit Zip™ as a measure of physical activity is recommended, according to the guidance of Welk et al. [13] This is supported by the finding that most of the participants favourably rated the utility of the Fitbit Zip™.

Physical activity monitors that allow individuals to upload their data to the internet may offer significant advantage over traditional devices, as they can integrate more easily into internet delivered physical activity interventions. Internet delivered interventions are an attractive mode of delivering public health interventions as they can be offered to large numbers of people at one time, at minimal cost [3].

It should however be noted that the Fitbit Zip™ records a significantly higher number of steps per day compared to the Actigraph. Differences in output from accelerometers and mechanical pedometers [15] and internet enabled pedometers [16] have been reported previously, and our findings suggest this is also true for the new generation of piezoelectric pedometers such as the Fitbit Zip™. Tudor-Locke et al. [15] concluded that these differences could arise from differences in instrument sensitivity thresholds or the method of attaching the device when wearing them. This suggests although the devices are reporting physical activity with the same units (steps/day), they are not equivalent and therefore caution should be exercised in future research seeking to combine information from accelerometers and pedometers in future analyses.

#### Strengths and limitations

The study participants were university employees, therefore validation in other population groups may be required. However the included participants undertook a wide range of physical activity levels (ranged from 3756 to 14050 steps/day), suggesting they are representative of the general population.

The manufacturers recommend that the Fitbit Zip™ can be used in a number of body placements such as shirt pocket, bra, pants pocket, belt, or waistband. This paper only includes validation of the Fitbit Zip™ worn on the waistband [17].

### Conclusion

The Fitbit Zip™ is a valid measure of free-living physical activity in healthy adults which offers the advantage of being able to wirelessly upload pedometer data to a website or mobile application. However, caution should be exercised when synthesising with accelerometer data in future research.
